# Primary oral melanoma: A histopathological and immunohistochemical study of 22 cases of Latin America

**DOI:** 10.4317/medoral.17588

**Published:** 2011-12-06

**Authors:** Bruno A B. de-Andrade, Víctor H. Toral-Rizo, Jorge E. León, Elisa Contreras, Román Carlos, Wilson Delgado-Azañero, Adalberto Mosqueda-Taylor, Oslei P. de-Almeida

**Affiliations:** 1 DDS, MSc. Oral Pathology Section, Department of Oral Diagnosis, Piracicaba Dental School, University of Campinas (UNICAMP), Piracicaba, São Paulo, Brazil; 2 DDS, PhD. Oral Pathology Section, Department of Oral Diagnosis, Piracicaba Dental School, University of Campinas (UNICAMP), Piracicaba, São Paulo, Brazil; 3 DDS, MSc. Centro Clínico de Cabeza y Cuello, Ciudad de Guatemala, Guatemala; 4 DDS. Centro Clínico de Cabeza y Cuello, Ciudad de Guatemala, Guatemala; 5 DDS, PhD. Departamento de Patología, Medicina y Cirugía Oral. Facultad de Estomatología. Universidad Peruana Cayetano Heredia, Lima, Perú; 6 DDS, MSc. Departamento de Atención a la Salud. Universidad Autónoma Metropolitana Xochimilco, México, D.F

## Abstract

Objective: The aim of this study was to analyze the histopathological and immunohistochemical characteristics of 22 cases of primary oral melanomas (OM). Study Design: Twenty two cases of primary oral melanoma were analyzed by description of their histopathological features and immunohistochemical study using the antibodies S-100, HMB-45, Melan-A and Ki-67. Results: The mean age was 58 years and 14 cases were female. The main affected sites were the hard palate, followed by the upper gingiva. Microscopically, 15 cases presented level III of invasion, 2 cases were amelanotic and 13 showed a mixed epithelioid and plasmacytoid or spindle cells composition. Some cases showed necrosis, perivascular and perineural invasion. S-100 and HMB-45 were positive in all cases, but 3 cases were negative for Melan-A. The proliferative index with Ki-67 was high, with labeling index ranging from 15.51% to 63% of positive cells. Conclusion: S-100 and HMB-45 are more frequently expressed than Melan-A in primary oral melanomas and these markers are helpful to confirm the diagnosis.

** Key words:**Oral melanoma, histopathology, immunohistochemistry.

## Introduction

Melanoma is formed by malignant melanocytes, which are cells derived from the neural crest that produce melanin (1). Over 90% of melanomas occur in the skin, but they may also arise from mucosal surfaces or at other sites wherein neural crest migrate (1,2). Primary oral melanomas are rare, comprising 0.4-1.8% of all melanomas and 0.5% of oral malignances (3-5). Primary OM is more common in the palate and gingiva of adult patients, and initially asymptomatic contributing to the delay of diagnosis (6-10). Clinical features vary, but the most common presentation is an initial dark blue or black irregular macule, turning later to an ulcerated black nodule (4,10-12).

As in other types of melanomas, the histology of OM is variable. Basically it is considered three patterns, in situ, invasive and mixed, the latter is the most common corresponding to about 55% of the cases (4,12). The tumor is formed by epithelioid, spindle and plasmacytoid tumor cells arranged in a sheet-like, organoid, alveolar, solid or desmoplastic configuration (4,5,12,13). Most primary OM are heavily pigmented, but some are amelanotic and immunohistochemistry can be helpful to confirm the diagnosis of these cases (14-25).

The aim of this study is to describe the histological characteristics and expression of S-100, HMB-45, Melan-A and Ki-67 in 22 cases of primary OM of Latin American patients.

## Material and Methods

**Figure 1:**
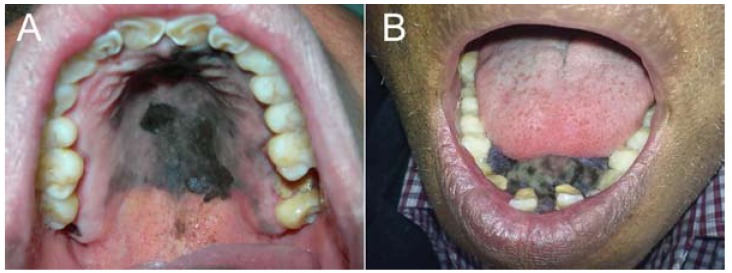
Clinical aspects of primary oral melanoma. A. Oral melanoma of the hard palate showing a large black, irregular macula with a small nodular area. B. Large pigmented melanoma of the lower gingiva causing tooth displacement.

Formalin-fixed, paraffin-embedded tissue blocks were obtained from 22 patients (8 men and 14 women, mean age 58 years, range of 23-86 years) with primary OM. Eleven cases were from Guatemala, 7 from Mexico, and 4 from Peru. Of the 22 OM, seven (31.82%) were located on the hard palate, five (22.73%) on hard palate and upper gingiva, four (18.18%) only on the upper gingiva, and two each (9.09%), on hard and soft palate, on the lower gingiva and on the buccal mucosa (Fig. 1).

Diagnosis of primary OM was confirmed by the clinical and histological characteristics, excluding the presence of melanoma at other anatomical sites and consequently the possibility of oral metastasis. The parameters evaluated included presence or absence of melanin in the tumor (melanotic or amelanotic), level of tumor cells invasion, cellular morphology (epithelioid, spindle, plasmacytoid or mixed) and pattern (solid, alveolar, organoid or pagetoid), necrosis, perineural and perivascular invasion.

Cellular invasion was considered according to Prasad et al. (26) as noninvasive (in situ), microinvasive (level I, cell clusters in the superficial lamina propria), invasive (level II, cell invasion into the lamina propria), and deep invasive (level III, invasion into skeletal muscle, bone or cartilage).

For immunohistochemical staining three-micrometer-thick sections were used. Briefly, after antigen retrieval with EDTA/Tris buffer (pH 9.0) in a microwave oven, endogenous peroxidase activity was blocked with 20% H2O2 for 5 cycles of 5 minutes each. Primary antibodies ( Table 1), after overnight incubation, were detected by secondary antibodies conjugated with polymer dextran marked with peroxidase (Dako EnVision Labelled Polymer; Dako, Glostrup, Denmark). The reaction was developed with Permanent Red (Permanent Red Substrate System, Dako) for the primary antibodies S-100, HMB-45 and Melan-A, and diaminobenzidine hydrochloride (DAB, Dako) was used as chromogen for Ki-67. The preparations were lightly counterstained with Carazzi hematoxylin, mounted with Aquatex (MERCK, Germany) and examined by light microscope.

**Table 1:**
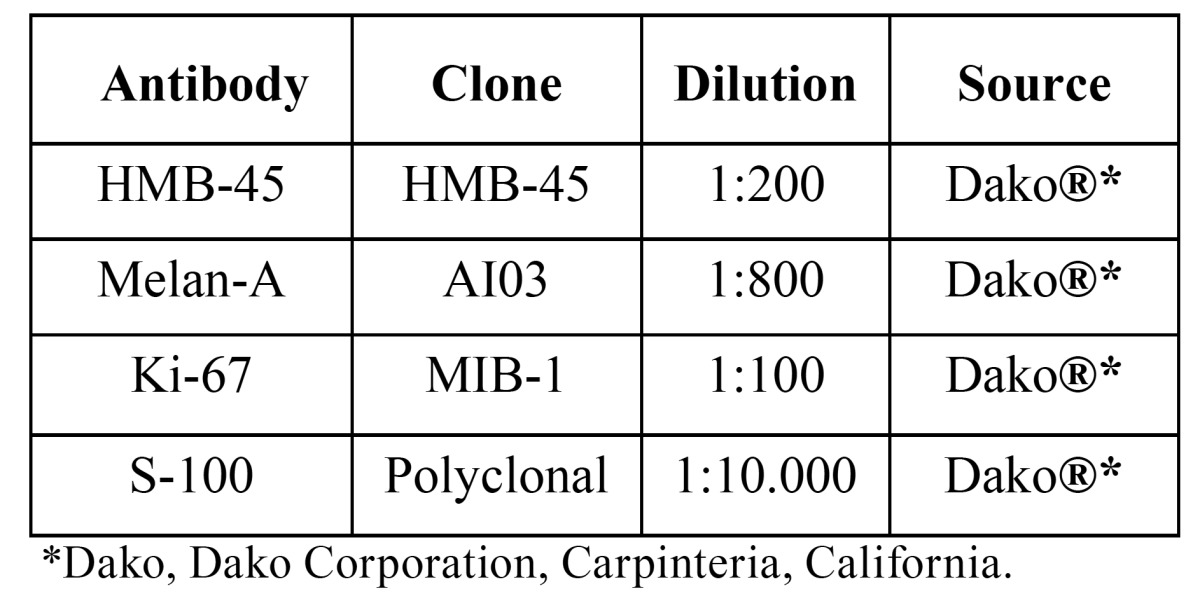
Antibodies used for immunohistochemical evaluation of 22 cases of primary oral melanomas.

The intensity of staining for S-100, HMB-45 and Melan-A was graded as follows: negative (-); weak (+) 1% to 25%; moderate (++) 25% to 50%; and strong (+++) 50% to 100% of the neoplastic cells. To calculate the labeling index (LI) for Ki-67, 1000 tumor cells were counted for each case and the results were expressed in percentage of positive cells.

## Results

**Figure 2:**
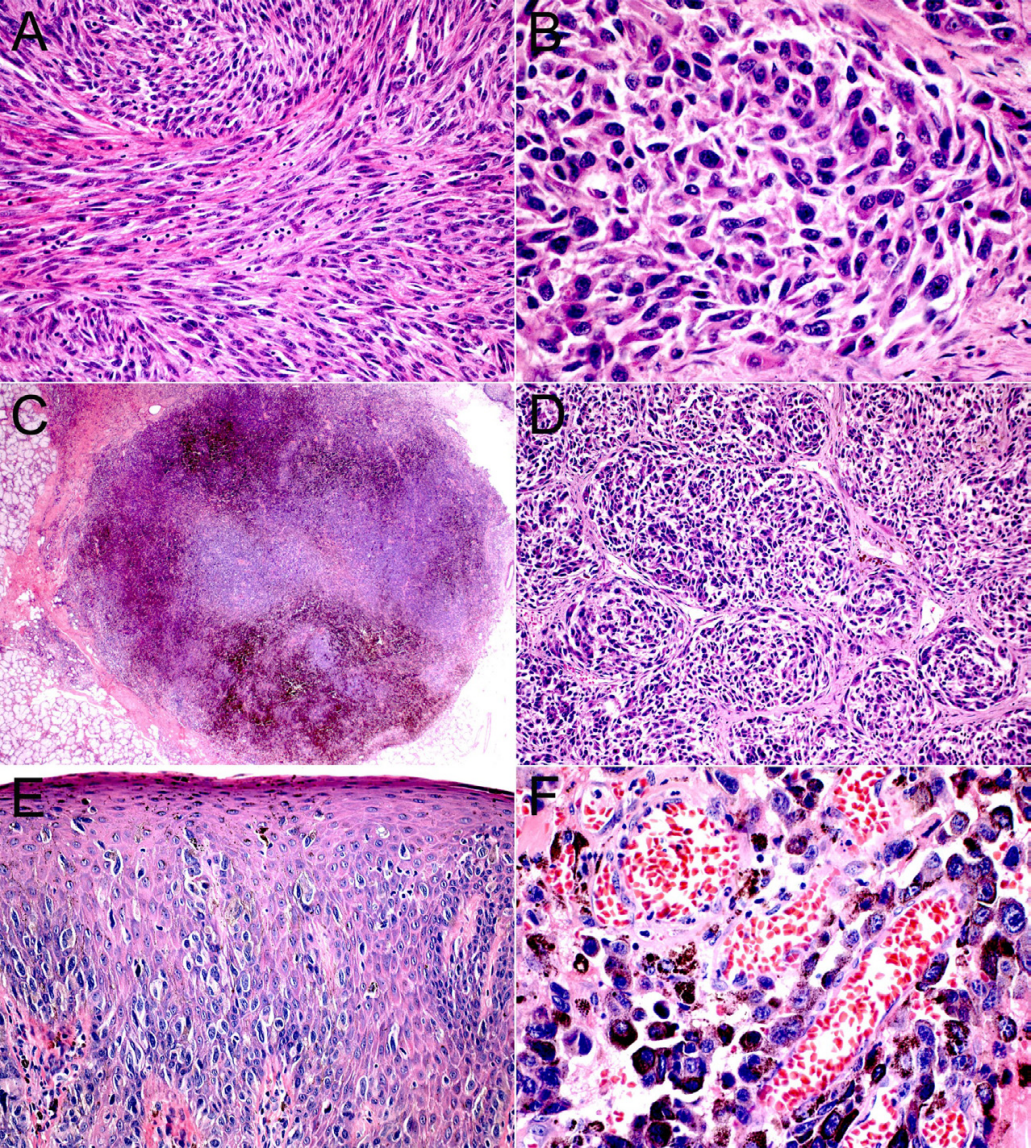
Histological aspects of primary oral melanoma (H&E). A. Oral melanoma of the hard palate composed mainly by amelanotic spindle cells (x200). B. Oral melanoma of the upper gingiva formed by epithelioid and plasmacytoid neoplastic melanocytes (x400). C. Solid OM showing nodular pattern of neoplastic melanocytic cells (x25). D. Epithelioid and plasmacytoid neoplastic melanocytes arranged in organoid pattern (x100). E. In situ lesion with neoplastic melanocytes arranged in pagetoid pattern (x100). F. Oral melanoma of the hard palate showing perivascular invasion (x400).

Twenty out of 22 primary oral melanomas studied were melanotic, and only two were amelanotic. Fifteen cases (68.18%) showed deep cellular invasion (level III) when diagnosed, while 4 and 2 cases presented levels II and I respectively, and only one case was classified as in situ. Cellular morphology varied, with 8 cases formed by epithelioid and one by spindle cells, while the other 13 cases showed a mixed population of epithelioid and plasmacytoid or spindle cells. Most cases showed a predominant cellular solid pattern (17 cases-77.27%), followed by alveolar (3 cases), with organoid and pagetoid pattern in only one case each. Necrosis, perivascular and perineural invasion was found in 6, 7 and 3 cases respectively (Fig. 2).

**Figure 3:**
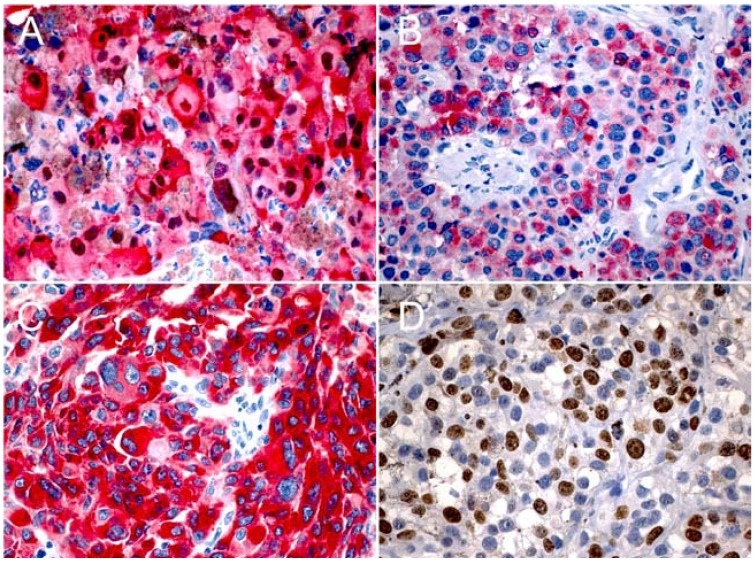
Expression of S-100 (A), HMB-45 (B), Melan-A (C), and Ki-67 (D) in primary oral melanoma of hard palate and upper gingiva (x400).

Immunostaining with three melanocytic markers S-100, HMB-45 and Melan-A in melanoma cells was cytoplasmic, but nuclear staining was also noted with S-100. S-100 and HMB-45 were expressed in all cases, while Melan-A was negative in 3 cases. S-100 was positive in more than 50% of tumor cells in all cases, and HMB-45 had a strong staining in 21 cases (95.45%), and in one it was moderate. Melan-A was expressed in 19 out of 22 cases; in 16, one and 2 cases it was strong, moderate and weak respectively. Cell proliferation with Ki-67 ranged from 15.51% to 63% of positive cells (Fig. 3).

## Discussion

Primary OM is a rare neoplasm accounting for only 0.5% of all oral malignances, with an incidence of 1.2 cases per 10 million per year (4,10,13). As it is very rare, there are few reported series of OM. It is considered that in Europe and Australia the incidence of OM is lower than in Japan and in other nonwhite populations like in Uganda, India and North American Indian (2,6-8). Series of OM cases in Latin America have been reported (13,23,24), but information of histological and immunohistochemical profile is scarce.

Different from the skin there is not a definitive clinicopathological classification of OM (10,12,23). Classification and staging of cutaneous melanomas using Breslow and Clark levels cannot be applied to oral mucosa tumors due to structural differences between skin and mucosa (13,25). In fact OM differ from cutaneous in several aspects, including risk factors as actinic radiation, a family history and association with atypical nevi, factors applied mainly to cutaneous melanoma (4). In our present study we used the histological classification proposed by Prasad et al. (26) based on the level of cellular invasion. As noted previously, in contrast to cutaneous melanomas where the majority are diagnosed in the radial growth phase, OM are found predominantly in the vertical growth phase (4,12). Most cases in our study were diagnosed with deep invasion (level III) and only one as in situ. Although lesions in the mouth usually are easily visualized, particularly if pigmented, most cases of OM are in advanced stages when diagnosed. Unfortunately, this is also true for oral squamous cell carcinoma, despite extensive efforts for early detection of this relatively common neoplasia. Our results were similar to those found by Lourenço et al. (13), with 82.35% of the cases diagnosed as level III, with only one case as level I. Oral melanomas have a poor prognosis, and according to Prasad et al. (26) the most important histopathological predictor is tumor histological level.

Presence of melanin was observed in 20 cases, as 2 were amelanotic. In fact, it is known that over 90% of oral melanomas contain melanin that can be easily observed in routine hematoxilin and eosin (5,27). Bachar et al. (28) studied 61 cases of head and neck mucosal melanoma finding 6 amelanotic cases (12.8%), similar to the values found in our study.

Different cell types, showing epithelioid, spindle and plasmacytoid morphology have been described in OM (5,12,13). In our series epithelioid cells predominated in most cases, either as a monomorphous or polymorphous infiltrate, usually mixed with plasmacytoid or spindle cells. The type of cellular infiltrate seems to have no impact in prediction of tumor behavior; however, Lourenço et al. (13) observed in their series that polymorphous tumors had a slightly higher incidence of vascular infiltration and necrosis. Primary OM are composed of nests or sheets of malignant cells in solid, alveolar or organoid patterns, as found in our series where the solid pattern (77.27%) predominated. As for the cell type, the cellular pattern also does not seem to influence the prognosis (5,12,13).

Vascular and perineural invasion was seen in 7 and 3 cases respectively, and similar results were described by Lourenço et al. (13), who identified 45.71%, 60.61% and 25.71% of cases with necrosis, perivascular and perineural invasion respectively. It is interesting that vascular invasion is more common than perineural, different from other more common oral tumors, as carcinomas and adenocarcinomas such as adenoid cystic carcinoma and polymorphous low-grade adenocarcinoma. Although not commonly observed, and therefore not considered to be an independent factor in most prognostic models, vascular invasion when present in cutaneous melanoma appears to be associated with a worse prognosis (14). As suggested by Prasad et al. (29) presence of vascular invasion potentially is an important indicator of poor prognosis in OM, as this is the first step for regional and distant metastases.

It is important to determine whether OM is a primary or metastatic lesion. Clinical and microscopical characteristics should be considered as site of involvement, presence or absence of pigmentation, overlying mucosal ulceration, extension along salivary glands ducts, and vascular and perineural invasion (4,6). Pigmented lesions involving the palate and gingiva, with ulcerated mucosa and extension along minor salivary gland are common findings in primary OM. On the other hand involvement of base of tongue with intact overlying mucosa, palatal and gingival sparing, vascular and perineural invasion are more commonly seen in metastatic melanoma (4,6).

It is well known that melanoma has a wide spectrum of histological features which mimic epithelial, hematological, mesenchymal and neural tumors, and when necessary immunohistochemistry is the primary tool to establish the correct diagnosis (14-16,30). Yu et al. (17) studied the expression of melanocytic differentiation markers in 6 primary OM. They showed that HMB-45, S-100 and Melan-A were positive in 100%, 83% and 67% of the cases respectively, suggesting that both HMB-45 and S-100 are good markers for immunohistochemical diagnosis of primary OM. In this study, we found that S-100 was a more sensitive marker than HMB-45 and Melan-A, although it is much less specific. On the other hand Prasad et al. (16) found that in sinonasal and OM the least sensitive marker was HMB-45, marking 85% of the cases, while S-100 and Melan-A were positive in about 95% of the cases. In cutaneous melanomas, S-100 is the most sensitive marker with expression in about 98% of cases, but again it is the least specific (14-17). HMB-45 and Melan-A show similar specificity in cutaneous melanomas with expression varying from 77-100% (14-17,20). Ki-67 staining is reported as positive in 13-30% of the cells in cutaneous melanoma, although individual cases can show almost 100% of nuclear positivity (14,15,31). In our study Ki-67 showed proliferating index ranging from 15.51% to 63% of positive cells. The present study did not consider treatment and prognosis of OM, but it is well established that treatment is surgical with a very poor prognosis, with 1 and 5 years survival of 75% and 15% respectively (4).

In summary, primary OM is rare, occurs in adult and elderly patients and usually are diagnosed at advanced stages, with very poor prognosis. It is formed by epithelioid, spindle, plasmacytoid or a mixture of these cells arranged in solid, alveolar and pagetoid pattern. S-100 and HMB-45 are more frequently expressed than Melan-A and these markers are helpful to confirm the diagnosis.

## References

[1] Femiano F, Lanza A, Buonaiuto C, Gombos F, Di Spirito F, Cirillo N (2008). Oral malignant melanoma: a review of the literature. J Oral Pathol Med.

[2] Hashemi Pour MS (2008). Malignant melanoma of the oral cavity: a review of literature. Indian J Dent Res.

[3] Gu GM, Epstein JB, Morton TH (2003). Intraoral melanoma: long-term follow-up and implication for dental clinicians. A case report and literature review. Oral Surg Oral Med Oral Pathol Oral Radiol Endod.

[4] Hicks MJ, Flaitz CM (2000). Oral mucosal melanoma: epidemiology and pathobiology. Oral Oncol.

[5] Meleti M, Leemans CR, Mooi WJ, Vescovi P, van der Waal I (2007). Oral malignant melanoma: a review of the literature. Oral Oncol.

[6] Rapini RP, Golitz LE, Greer RO Jr, Krekorian EA, Poulson T (1985). Primary malignant melanoma of the oral cavity. A review of 177 cases. Cancer.

[7] Wagner M, Morris CG, Werning JW, Mendenhall WM (2008). Mucosal melanoma of the head and neck. Am J Clin Oncol.

[8] Manolidis S, Donald PJ (1997). Malignant mucosal melanoma of the head and neck: a review of the literature and report of 14 patients. Cancer.

[9] Umeda M, Komatsubara H, Shigeta T, Ojima Y, Minamikawa T, Shibuya Y (2008). Treatment and prognosis of malignant melanoma of the oral cavity: preoperative surgical procedure increases risk of distant metastasis. Oral Surg Oral Med Oral Pathol Oral Radiol Endod.

[10] Garzino-Demo P, Fasolis M, Maggiore GM, Pagano M, Berrone S (2004). Oral mucosal melanoma: a series of case reports. J Craniomaxillofac Surg.

[11] Younes MN, Myers JN (2004). Melanoma of the head and neck: current concepts in staging, diagnosis, and management. Surg Oncol Clin N Am.

[12] Barker BF, Carpenter WM, Daniels TE, Kahn MA, Leider AS, Lozada-Nur F (1997). Oral mucosal melanomas: the WESTOP Banff workshop proceedings. Western Society of Teachers of Oral Pathology. Oral Surg Oral Med Oral Pathol Oral Radiol Endod.

[13] Lourenço SV, A MS, Sotto MN, Bologna SB, Giacomo TB, Buim ME (2009). Primary oral mucosal melanoma: a series of 35 new cases from South America. Am J Dermatopathol.

[14] Banerjee SS, Harris M (2000). Morphological and immunophenotypic variations in malignant melanoma. Histopathology.

[15] Ohsie SJ, Sarantopoulos GP, Cochran AJ, Binder SW (2008). Immunohistochemical characteristics of melanoma. J Cutan Pathol.

[16] Prasad ML, Jungbluth AA, Iversen K, Huvos AG, Busam KJ (2001). Expression of melanocytic differentiation markers in malignant melanomas of the oral and sinonasal mucosa. Am J Surg Pathol.

[17] Yu CH, Chen HH, Liu CM, Jeng YM, Wang JT, Wang YP (2005). HMB-45 may be a more sensitive maker than S-100 or Melan-A for immunohistochemical diagnosis of primary oral and nasal mucosal melanomas. J Oral Pathol Med.

[18] Moore BW (1965). A soluble protein characteristic of the nervous system. Biochem Biophys Res Commun.

[19] Salama I, Malone PS, Mihaimeed F, Jones JL (2008). A review of the S100 proteins in cancer. Eur J Surg Oncol.

[20] Busam KJ (2004). The use and application of special techniques in assessing melanocytic tumours. Pathology.

[21] Gown AM, Vogel AM, Hoak D, Gough F, McNutt MA (1986). Monoclonal antibodies specific for melanocytic tumors distinguish subpopulations of melanocytes. Am J Pathol.

[22] Carlson JA, Ross JS, Slominski AJ (2009). New techniques in dermatopathology that help to diagnose and prognosticate melanoma. Clin Dermatol.

[23] Lopez-Graniel CM, Ochoa-Carrillo FJ, Meneses-García A (1999). Malignant melanoma of the oral cavity: diagnosis and treatment experience in a Mexican population. Oral Oncol.

[24] Delgado-Azañero WA, Mosqueda-Taylor A (2003). A practical method for clinical diagnosis of oral mucosal melanomas. Med Oral.

[25] Chidzonga MM, Mahomva L, Marimo C, Makunike-Mutasa R (2007). Primary malignant melanoma of the oral mucosa. J Oral Maxillofac Surg.

[26] Prasad ML, Patel SG, Huvos AG, Shah JP, Busam KJ (2004). Primary mucosal melanoma of the head and neck. A proposal for microstaging localized, stage I (lymphnode-negative) tumors. Cancer.

[27] Sortino-Rachou AM, Cancela Mde C, Voti L, Curado MP (2009). Primary oral melanoma: Population-based incidence. Oral Oncol.

[28] Bachar G, Loh KS, O’Sullivan B, Goldstein D, Wood S, Brown D (2008). Mucosal melanomas of the head and neck: experience of the Princess Margaret Hospital. Head Neck.

[29] Prasad ML, Patel S, Hoshaw-Woodard S, Escrig M, Shah JP, Huvos AG (2002). Prognostic factors for malignant melanoma of the squamous mucosa of the head and neck. Am J Surg Pathol.

[30] Demierre MF, Sabel MS, Margolin KA, Daud AI, Sondak VK (2008). State of the science 60th anniversary review: 60 Years of advances in cutaneous melanoma epidemiology, diagnosis, and treatment, as reported in the journal Cancer. Cancer.

[31] Frahm SO, Schubert C, Parwaresch R, Rudolph P (2001). High proliferative activity may predict early metastasis of thin melanomas. Hum Pathol.

